# Surface Roughness Influence on Néel-, Crosstie, and Bloch-Type Charged Zigzag Magnetic Domain Walls in Nanostructured Fe Films

**DOI:** 10.3390/ma13194249

**Published:** 2020-09-24

**Authors:** Cristina Favieres, José Vergara, Vicente Madurga

**Affiliations:** 1 Laboratory of Magnetism, Department of Science. Physics, Public University of Navarre, Campus Arrosadía s/n, E-31006 Pamplona, Spain; jvergara@unavarra.es (J.V.); vmadurga@unavarra.es (V.M.); 2 Institute for Advanced Materials and Mathematics, INAMAT2, Public University of Navarre, Campus Arrosadía s/n, E-31006 Pamplona, Spain

**Keywords:** magnetic thin films, charged domain walls, magneto-optic, roughness energy, magnetic wall energy, pulsed laser deposition (PLD), scanning tunneling microscopy (STM)

## Abstract

Charged magnetic domain walls have been visualized in soft magnetic nanostructured Fe thin films under both static and dynamic conditions. A transition in the core of these zigzagged magnetic walls from Néel-type to Bloch-type through the formation of crosstie walls has been observed. This transition in charged zigzagged walls was not previously shown experimentally in Fe thin films. For film thicknesses *t* < 30 nm, Néel-type cores are present, while at *t* ≈ 33 nm, walls with crosstie cores are observed. At *t* > 60 nm, Bloch-type cores are observed. Along with the visualization of these critical parameters, the dependence on the film thickness of the characteristic angle and length of the segments of the zigzagged walls has been observed and analyzed. After measuring the bistable magneto-optical behavior, the values of the wall nucleation magnetic field and the surface roughness of the films, an energetic fit to these nucleation values is presented.

## 1. Introduction

Magnetic thin films have long been a subject of study from both fundamental and applied points of view because of the special properties that make them appropriate for numerous technical devices, including sensors and actuators [1,2]. In particular, Fe and Fe-based films are attractive for these purposes because of their high saturation magnetization at room temperature, high magnetostriction, and controllable magnetic anisotropy through the appropriate selection of deposition techniques and conditions, such as growth temperature, type of substrate, underlayer, capping layer, incident plasma angle, and thickness [3,4,5,6]. These parameters also allow researchers to control certain properties, such as interfacial diffusion [7], interface roughness [8], and possible strain due to lattice mismatch between films and substrates [9], all of them having a noticeable influence on the magnetic properties. Fe-based films have also demonstrated their suitability for magnetic tunnel junctions and spintronic applications [10,11,12]. In particular, in previous studies we showed that nanostructured Fe thin films obtained by pulsed laser deposition (PLD), either planar or cylindrically shaped, exhibited high room temperature saturation magnetization and high magnetostriction as well as suitable responses for ultra-high-frequency applications [13,14,15].

Magnetic devices that can be controlled by their magnetic domain structures and separating walls have been developed [16]. Magnetic walls are often used as the key parameters in non-volatile memory devices [17]. Comprehensive knowledge of the magnetic domain structures is also necessary from an applied point of view [18], as domain structure investigations provide information about the energetic balance among the interactions that stabilize the ferromagnetic structure, such as magnetostatic, exchange, and anisotropy energy. So, the study of magnetic domain structures is important, and they are continuously the object of analysis and research [19,20,21,22,23]. Indeed, the control of magnetic domain walls in differently shaped materials such as thin films [24], nanoparticles [25], nanowires [26], nanodots [27], etc., is the subject of numerous investigations for the development of functional materials for scientific and technological applications.

Charged walls are special types of magnetic domain walls that can occur in thin films [28]. These walls, appearing as zigzag or “saw-tooth” walls, form between domains of opposite head-on magnetization directions displaying a characteristic vertex angle of θ. The zigzag reduces the magnetostatic energy, so the charge density decreases and the length of the wall increases. The particular shape of the wall is determined by the minimization of total energy. According to the spine model, charged walls can exhibit a specific configuration to reduce stray field energy associated with the net charge: they form a long-range tail (a tail of Néel walls) and a spine [28,29,30]. The Néel tail transition involves an in-plane magnetization rotation corresponding to two adjacent magnetic moment carriers. The region surrounding the spine or core exhibits a non-uniform magnetization ***M****,* and there is a non-zero divergence of *M*, ∇**M** ≠ 0, resulting in the presence of magnetic poles in this region. Depending on the film thickness, this wall’s spine must correspond to Bloch, crosstie, or Néel walls [28,30]. Several models have dealt with micromagnetic calculations of the magnetization distribution within zigzag walls, and some models have also included the dynamic hysteresis from such walls [30,31,32]. Charged zigzag domain walls have been observed in different systems, such as CoGd and CoGdNi [33], including, more recently, pulsed laser-deposited Co thin films [34,35] (and references therein), epitaxial Fe films grown on GaAs [36], permalloy/niobium bilayers [37], SmCo amorphous films [38], FePt thin films [39], CoFeB antiferromagnetically coupled layers [40], ultrathin yttrium iron garnet/Pt bilayers [41], single crystals of ferromagnetic shape memory Co_50_Ni_20_FeGa_29_ alloy [42], and highly anisotropic CoFeB thin films [43]. However, to date, no experimental research has shown the transition from Néel- to Bloch-type cores with the formation of crosstie cores in Fe-charged domain walls.

In this work we present a study of zigzag charged magnetic domain walls in nanostructured soft magnetic Fe thin films. We provide the first experimental evidence of the crossover between Néel-type and Bloch-type cores of these charged walls in Fe films. We demonstrate the predicted [28,30] presence of crosstie cores as a transition between the Néel and Bloch cores. We show the dependence on the film thickness of the characteristic angle and the length of the segments of zigzag walls. After measuring the bistable magneto-optical behavior and surface roughness of the samples, the values of the wall nucleation magnetic field were fitted according to the magnetic energy involved in these structures, showing the importance of the surface roughness.

## 2. Materials and Methods 

Fe films were prepared by pulsed laser deposition (PLD). This technique is a suitable and versatile method of preparing an extensive range of thin films, including magnetic films [13,14,15,34,35,44,45,46,47,48,49,50,51,52]. We used a chamber (Neocera, Beltsville, MD, USA) at a base vacuum pressure of 10^–6^ mbar. A 99% pure, 25 mm diameter Fe disk (Goodfellow, Huntingdon, UK) was prepared as the target. It was mechanically polished before each ablation process. During PLD, the target rotated around its axis of cylindrical symmetry at a constant angular speed of 32 rpm. A pulsed Nd:YAG laser beam (Quantel Brilliant, Les Ulis, France) (λ = 1024 nm, 20 Hz repetition rate, 4 ns pulses, energy per pulse of 220 mJ just on the target (laser fluence ≈ 1.7 J cm^−2^)) was introduced into the chamber through a quartz window. The laser beam’s area on the target was approximately 13 mm^2^ and incidence angle on the target plane was 45°. Before deposition, the polished target was ablated for 1 min to remove surface contaminants. Different substrates were used for structural studies: rectangular pieces of glass and 25 × 10 mm^2^ rectangular pieces of single crystalline Si (111). All of the substrates were cleaned in pure methanol prior to deposition. We used a normal deposition, with the substrate surface perpendicular to the ejected plasma’s direction. The substrates were 75 mm from the target and at room temperature. Under these conditions, the deposition rate measured with a 6 MHz quartz crystal oscillator was 11 nm min^–1^. The deposition time varied to obtain samples with different thicknesses between 10 and 110 nm. A custom-made substrate holder allowed the substrates to rotate at 120 rpm around an axis perpendicular to the substrate’s plane to minimize possible anisotropy caused by the non-uniform distribution of the laser beam’s energy. We used the following deposition parameters and conditions: Nd:YAG laser with its fundamental wavelength, particular fluence, glass and Si substrates, target-to substrate distance, normal incidence, and deposition time; from our previous works and from other studies [34,35,44,45,50,51,52], because we knew that they allow the controlled growth of nanostructured (grain size around 1–2 nm) soft magnetic films.

Structural studies were conducted using x-ray diffraction (Seifert diffractometer, Ahrensburg, Germany) in grazing incidence mode (incidence angle of 0.5°, 2θ scan between 15 and 40° in steps of 0.04°, and counting time of 32 s/step) with Mo Kα radiation (λ = 0.71073 Å) operating at 50 kV and 40 mA.

The magnetic microstructures of the films grown over the glass substrates were observed by the Bitter technique using a colloidal suspension (Ferrofluidics EMG707) [34,35,53,54,55,56,57]. The fine magnetic particles of the suspension are attracted to regions of higher gradient corresponding the non-uniform magnetic field created by the film, so they deposit as a band along the edge of the domain, revealing the magnetic walls [58] (p. 285). This allows the examination of a very large area of the sample surface and is quite sensitive to small variations in magnetization. We examined the magnetic structure corresponding to the sample’s 10 × 6 mm^2^ central region. When it was necessary to improve the contrast and pattern resolution, we applied a magnetic field (1270 A m^−1^) perpendicular to the sample surface to polarize the colloidal suspension. All images were digitally cleaned and enhanced.

Magnetic hysteresis loops of the films were measured at room temperature using the transverse magneto-optical Kerr effect (MOKE), based on the interaction of light and matter [59]. The measurements are based on the change of polarization plane of a linearly polarized light beam when it is reflected by the surface of a magnetic sample situated in a magnetic field. This change produces a change in the intensity of the reflected light with respect to the incident light, which is proportional to the magnetization of the sample. A custom-built system allowed rotation of the samples around an axis perpendicular to the film plane inside the AC (18 Hz) in-plane magnetic field (created using a pair of Helmholtz coils) to find possible easy in-plane magnetization directions. The spotlight fell upon the center of the sample’s 10 × 6 mm^2^ central region.

The surface nanomorphology of the samples was studied by scanning tunneling microscopy [60] (STM, Burleigh, Fishers, NY, USA). The images were processed by WSxM free software from Nanotec [61].

## 3. Results and Discussion

As pointed out above, this work is focused on the study of charged magnetic domain walls of Fe films, and the main purpose is to show the first experimental evidence of the crossover between Néel-type and Bloch-type cores of these Fe-charged walls with the presence of crosstie walls, which was predicted some time ago. Taking into account the different magnetic energies involved in these structures, an energetic fit to the values of the nucleation magnetic field is presented.

To this end, we show first the experimental results obtained on the structure of the Fe films and their magnetization processes. We focus particularly on the values of the nucleation magnetic field of the magnetic walls obtained from the corresponding hysteresis loops. Second, we show the charged magnetic domain wall structures. Following that, images of the surface roughness of the films are shown. A discussion covers an energetic study to explain the observed behavior.

### 3.1. Film Structure

**Figure 1:**
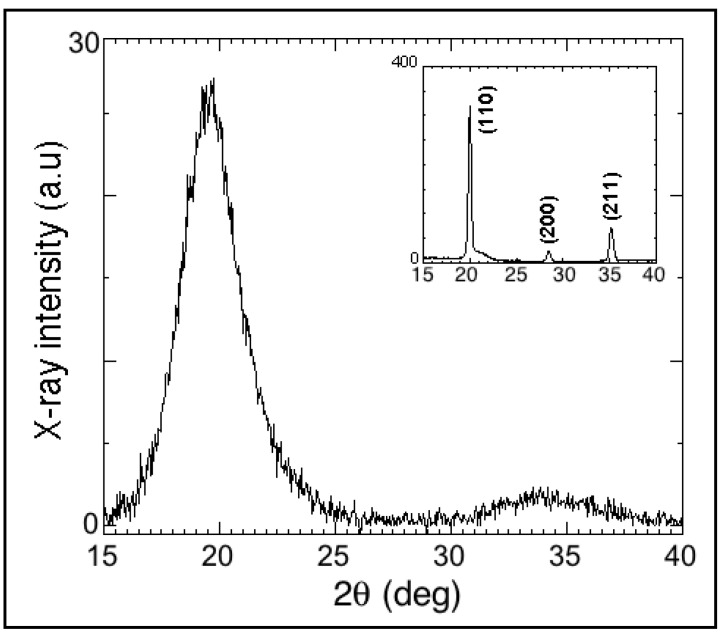
X-ray diffractogram corresponding to as-deposited pulsed laser deposition (PLD) Fe film showing the typical halo of amorphous or nanocrystalline material. Inset shows diffractogram corresponding to crystalline Fe target used for deposition, where crystalline peaks corresponding to (110), (200), and (211) planes were detected.

Figure 1 shows the x-ray diffractogram characteristics of one of our 110 nm thick as-deposited Fe films. It reveals that no peaks specific to a crystal structure were present, exhibiting the halo characteristics of amorphous or nanocrystalline materials. The nanocrystal size estimated using Scherrer’s formula was ≈1.5 nm.

### 3.2. Magneto-Optical Behavior

**Figure 2:**
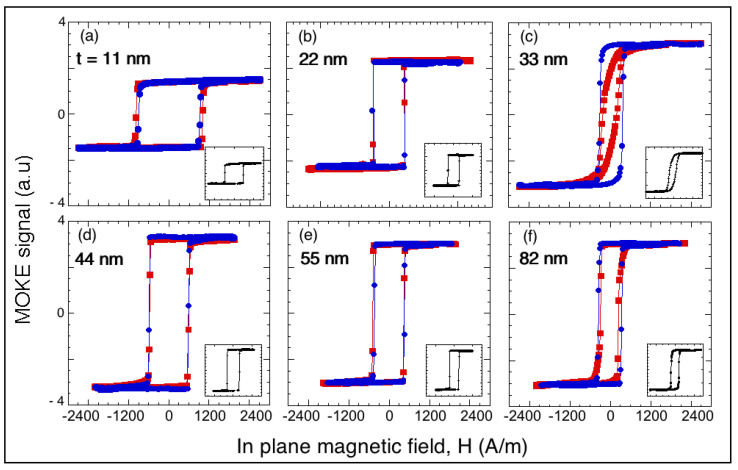
(**a**–**f**) Magneto-optical Kerr effect (MOKE) hysteresis loops corresponding to as-deposited Fe films with different thickness *t*. Films were successively magnetized along two mutually perpendicular in-plane directions defined as *L* (square red symbols) and *T* (circular blue symbols). Insets show loops obtained when films were magnetized at 45° from the *L* and *T* directions. For all samples, the three loops were very similar, reinforcing their in-plane isotropic magnetic properties. Horizontal and vertical scales are the same for all of graphics, including insets; lines are visual guides.

Figure 2 shows the MOKE hysteresis loops corresponding to films with different thicknesses deposited on the glass substrates. The magnetic field was applied in the films’ plane. The loops were measured in two mutually perpendicular directions, labeled *L* (along the rectangular film’s long direction) and *T* (along the short direction). The insets show the loops obtained when measuring at 45° to the *L* and *T* directions. Soft magnetic characteristics, bistable behavior, and the absence of in-plane magnetic anisotropy in most of the films was deduced from these loops. The similar behavior of the three in-plane hysteresis loops for each sample, shown in Figure 2, revealed their in-plane isotropic magnetic properties. Only the 33 nm thick film shown in Figure 2c displayed different behavior than the other samples. With the exception of this sample, all of the films displayed maximum squareness in the three directions, indicating that domain wall nucleation was the dominant reversal mechanism.

**Figure 3:**
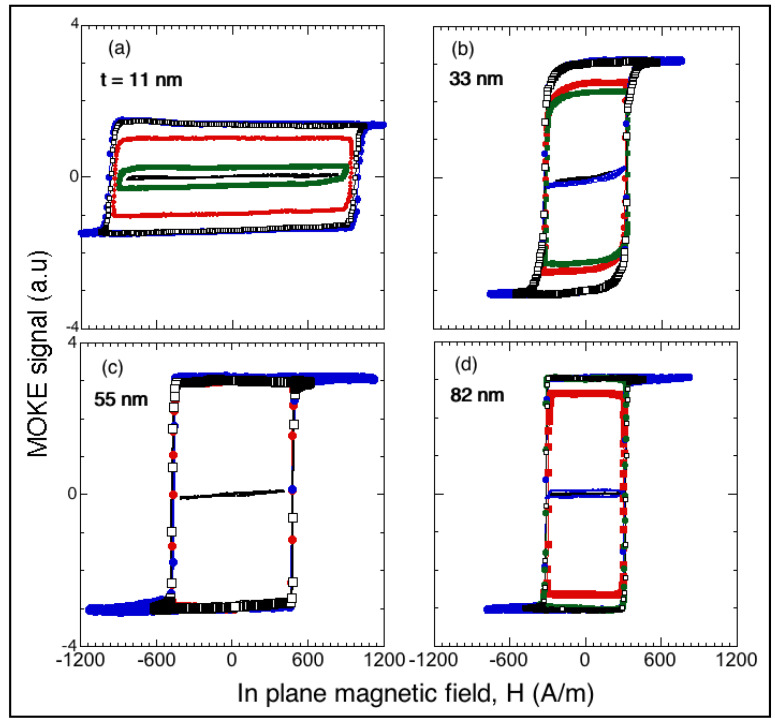
(**a**–**d**) MOKE hysteresis loops along the *T* direction corresponding to as-deposited Fe films with different thicknesses *t* showing different nucleation magnetic field values *H*_N_ depending on *t*. Loops were obtained by applying magnetic fields of increasing strength so that irreversible nucleation and immediate displacement of magnetic walls could be precisely observed. Lines are visual guides.

Figure 3 shows the MOKE loops measured at lower fields than those corresponding to the loops in Figure 2; they confirmed the magnetization processes as irreversible nucleation and immediate displacement of the magnetic walls. As shown Figure 3a, a very small difference was detected only in the thinnest sample, 11 nm, between nucleation magnetic field *H*_N_ = 900 A m^–1^ and coercive field *H*_C_ = 955 A m^–1^, probably because of the influence of surface roughness, which will be discussed later. As demonstrated in Figure 3b, the *t* = 33 nm film displayed behavior that was not observed in the other samples, because the loops were slightly rounded at fields very close to the saturation magnetization, as shown in Figure 2.

**Figure 4:**
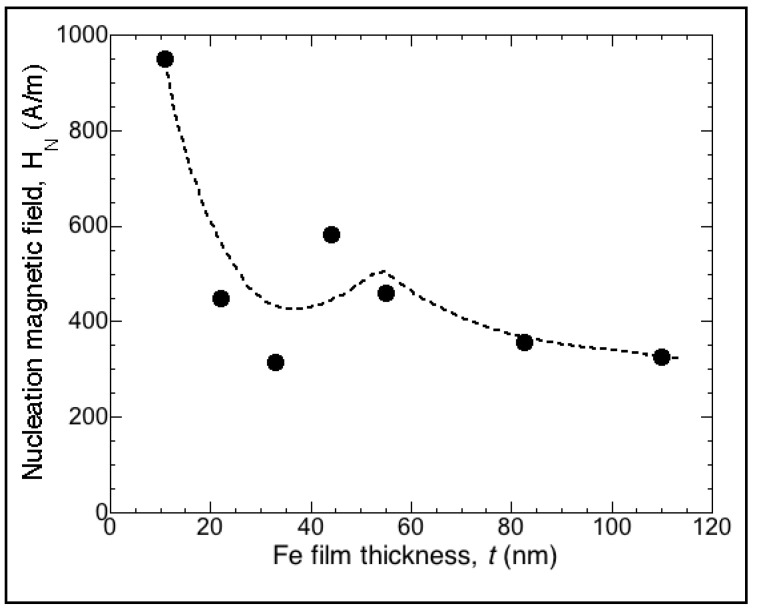
Dependence of nucleation magnetic field *H*_N_ on film thickness *t*. Overall behavior indicated that *H*_N_ decreased with thickness, although a slight increase was observed at thicknesses between ≈35 and 45 nm. Dashed line is a visual guide.

As demonstrated Figure 2 and Figure 3, the nucleation magnetic field *H*_N_ had values characteristic of soft magnetic materials and exhibited a dependence on film thickness *t*. The overall behavior indicated that *H*_N_ decreased with the thickness, although an increase in *H*_N_ was observed at thicknesses between ≈35 and 45 nm (see Figure 4), which will be addressed in the Discussion section.

### 3.3. Magnetic Domain Wall Structure

**Figure 5:**
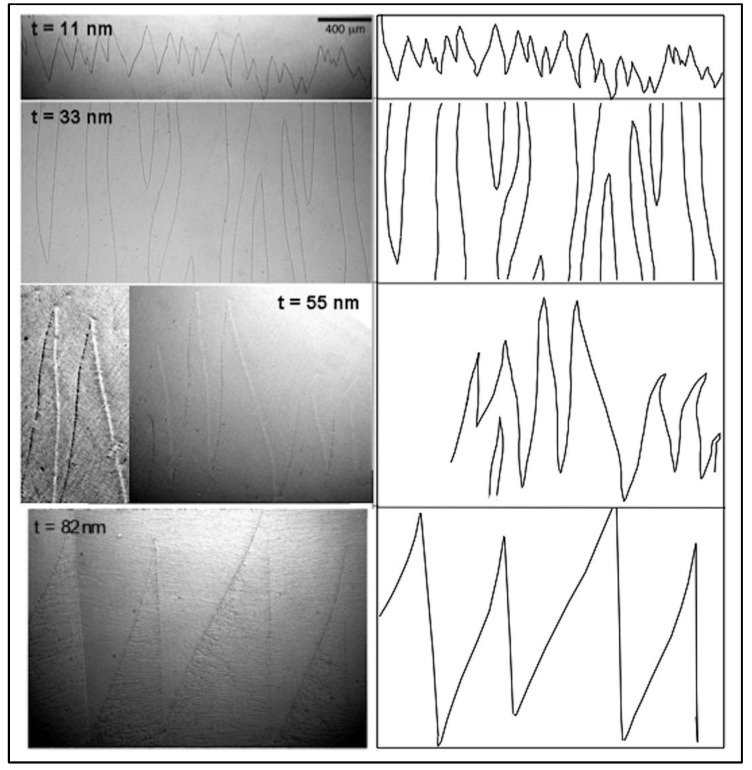
(**Left**): Bitter patterns of Fe films with different thicknesses *t*: charged zigzag walls are shown. Images were obtained while demagnetizing the films and were digitally cleaned and enhanced; additional image was superimposed on the left of the 55 nm film. (**Right**): Illustration of walls. Depending on sample thickness, charged walls exhibit different characteristic vertex angle, shape, lengths of segments of zigzag walls, and core type (see text for explanation).

Figure 5 shows Bitter micrographs corresponding to the magnetic domain structures of the Fe films with different thicknesses *t*. The walls were visible as zigzag walls.

As shown in Figure 5, in the thinnest film, *t* = 11 nm, the overall pattern of the charged domain walls was zigzag but did not have the non-straight shape of the wall segments. The film thinness and principally its negligible in-plane uniaxial anisotropy permitted local deviations in the direction of the magnetic moments, which were probably produced by the surface roughness, which generated a high value of the ratio *R*_q_/*t* [35], where *R*_q_ is the root mean square of surface roughness. This will be discussed in more detail later. In fact, there was more influence of the shape effect in the direction of the magnetic moments at the film surface produced by the surface roughness than on the magnetic moments of the film volume, which in this case was negligible due to the low *t* value. The exchange constant’s high value (A ≈ 2.1 × 10^–11^ J m^−1^ for crystalline Fe) [62] (p. 242) was overcome by the *R*_q_/*t* value. Due to the thickness of the film, *t* = 11 nm, the observed walls probably had a Néel-type core, similar to other magnetic thin films [35].

As the thickness increased to *t* = 33, 55, and 82 nm, the zigzag segments gradually became more regular in shape, as shown in Figure 5.

**Figure 6:**
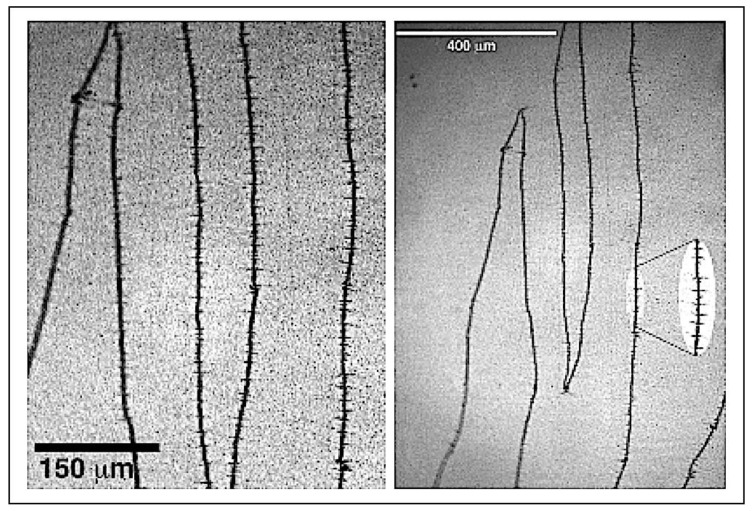
(**Left**): Details of magnetic microstructure of Fe film with *t =* 33 nm. Crosstie walls were observed within zigzag walls. (**Right**): Consecutive peaks corresponding to magnetic domain walls can be observed due to different image scales.

Of note, there was a change in the nature of the wall core at *t* ≈ 30 nm, as demonstrated in Figure 5 (*t* = 33 nm): a “crosstie” pattern appeared on the long magnetic walls. Figure 6 shows magnifications of these walls, emphasizing the visualization of the magnetic colloidal particles attracted by the crosstie segments clearly present along the main longitudinal magnetic walls. A higher density of magnetic colloidal particles in the proximity of the peaks of the walls was observed; the possible presence of two crosstie segments at these points produced an increase in the magnetic pole density in these vertices. The average value of the crosstie spacing was ≈12–15 μm.

As the thickness increased to 55 nm, the magnetic microstructures changed: a change in the contrast of two adjacent segment walls occurred as they appeared alternatively white and black, indicative of the presence of Bloch lines in the zigzag domain walls (digitally enhanced on the left side of the image for *t* = 55 nm in Figure 5) [63,64]. Shape irregularities were not present in the films with *t* = 55 nm. A very weak crosstie pattern was observed on the wall segments. The approximate average value of the crosstie spacing was ≈8–10 μm, lower than the 12–15 μm corresponding to the 33 nm thick sample. This increase in crosstie density was coincident with the increased wall nucleation field that we measured in a range of thickness between ≈ 35 and 45 nm.

By further increasing the film thickness, that is, *t* = 82 nm, the zigzag segments of the magnetic domain walls were straight, and only one sign of the magnetic poles was observed, as shown in Figure 5 (bottom). No crosstie was observed at this thickness, nor at higher *t*.

**Figure 7:**
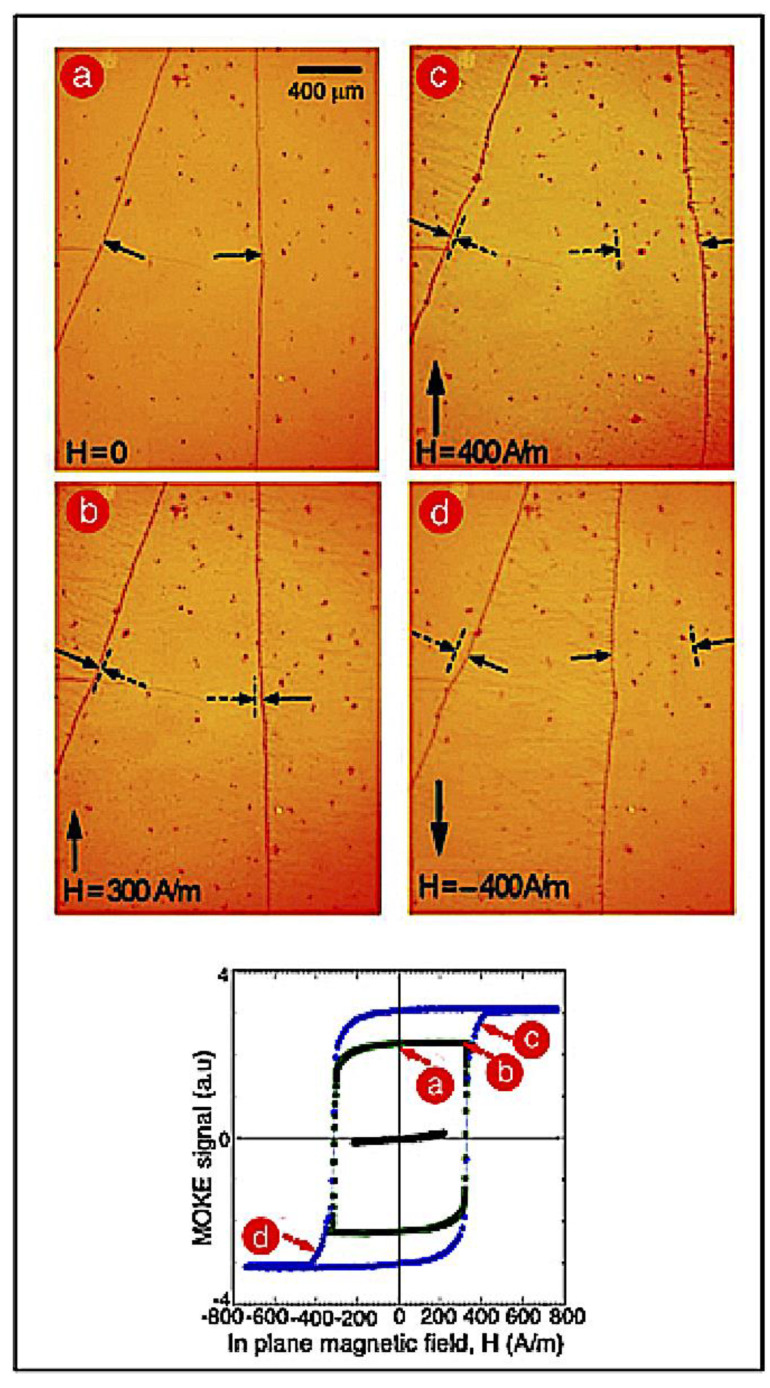
Movement of magnetic walls in 33 nm thick film when DC magnetic field was applied (large vertical arrows): (**a**) *H*_appl._ = 0, (**b**) *H*_appl._ = +300 A m^−1^, (**c**) *H*_appl._ = +400 A m^−1^, and (**d**) *H*_appl._ = −400 A m^−1^, demonstrating applied field direction. Small arrows in each image indicate position of magnetic wall before (dashed) and after (solid) application of magnetic field. **Bottom**: MOKE hysteresis loops at different applied field strengths indicating magnetic state in which images were obtained.

Figure 7 shows the movement of the magnetic walls with the 33 nm thick film when a DC magnetic field was applied. A field of 300 A m^–1^, as shown in Figure 7b, almost did not move the walls. An increase in the field to 400 A m^–1^ promoted wall displacements over a few hundred microns; the movement was observed in two opposite directions, as shown in Figure 7c,d, corresponding to the two opposite directions of the applied field. Figure 7 includes some MOKE loops of this sample showing the magnetization processes at various applied magnetic field strengths: it again confirmed the magnetization processes as nucleation and later displacement of the magnetic walls. The roundness of the loops near saturation could be attributed to the presence of the crosstie segments at 90° in the main longitudinal walls.

In summary, different types of charged magnetic domain walls were observed in the Fe films depending on their thickness.

(1) At thickness lower than ≈30 nm, the charged walls had Néel-type cores and exhibited irregular shapes.
(2) At thickness between ≈30 nm and 50 nm, the cores of the charged magnetic walls evolved to crosstie types and exhibited fairly straight forms.
(3) At thickness ≈ 55 nm, Bloch lines with very weak crosstie walls developed in the charged walls, alternating dark and bright zigzag segments.
(4) At thickness greater than 60 nm, Bloch-type core charged walls were visualized in the films. Long straight segments were observed in the zigzag walls of these films.
(5) Increased length of the corresponding segments of each zigzag wall *t* = 11 nm, 55 nm, and 82 nm was also observed, as demonstrated in Figure 5.

By further examining the structures shown in the Bitter patterns, in addition to the manifest change in the walls’ core type, the shape, vertex angle θ, length of zigzag segments, and period of zigzag walls differed from one sample to another depending on the film thickness.

### 3.4. The Surface Roughness

**Figure 8:**
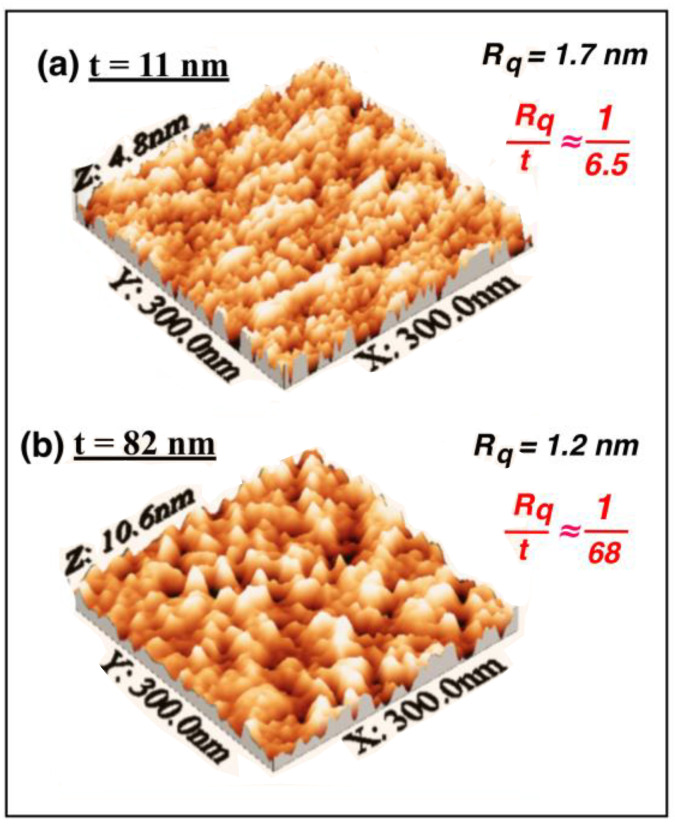
Some representative STM images corresponding to two different films, (**a**) *t* = 11 nm and (**b**) *t* = 82 nm. The surface roughness *R*_q_, the root mean square of the surface roughness, was similar for both, and *R*_q_/*t* ≈ 1/6.5 at *t* = 11 nm, *R*_q_/*t* ≈ 1/68 at *t* = 82 nm.

The surface roughness of the samples was measured. *R*_q_, the root mean square of the surface roughness, had mean values of 1.7 nm for the thinner *t* films (*t* = 11 nm), 1.5 nm for *t* = 33 nm, 2.0 nm for *t* = 55 nm, and 1.2 nm for the thicker *t* films (*t* = 82 nm). These are average values obtained when measuring at different scales (from 300 × 300 nm^2^ to 3 × 3 μm^2^). The corresponding values of the roughness-to-thickness ratio were *R*_q_/*t* ≈ 1/6.5 at *t* = 11 nm, *R*_q_/*t* ≈ 1/22 at *t* = 33 nm, *R*_q_/*t* ≈ 1/28 at *t* = 55 nm, and *R*_q_/*t* ≈ 1/68 at *t* = 82 nm. Figure 8 shows some representative STM images corresponding to two different films.

### 3.5. Discussion 

It is well known and established that in general, the energy balance for domain wall formation in ferromagnetic materials must consider the exchange energy, anisotropy energy and magnetostatic energy [65]. The last term is thickness dependent, so the wall structure depends on the film thickness. The magnetization rotation within the wall is produced in the film plane (Néel wall) if its thickness is comparable to the wall width, because in-plane rotation leads to lower wall energy compared to out-of-plane rotation (Bloch wall). In Néel walls, the energy increases if the film thickness increases, while in Bloch walls, the energy decreases with increasing thickness [28,58] (and references therein). When the film thickness increases, a transition from one type of domain wall to the other occurs in many cases, in the form of crosstie walls in which both types of rotation coexist. The energy of the crosstie walls is lower than that corresponding to Néel walls and is also related to the crosstie spacing [28].

It was also established that by alternating the rotation of the wall’s magnetic moments, the charge sign of the film surface alternates, also reducing the magnetostatic energy, as occurs in so-called Bloch lines [63,64].

In thin films with zigzag or saw-tooth magnetic walls, an additional magnetostatic self-energy term must be considered due to their Néel charged tails [28,30,66]. In addition, if the thin films are rough, extra energy accounting for the interactions of the magnetic moments with surface irregularities must be considered [35]. Indeed, in these films it must be taken into account that for lower thickness, the surface roughness contributes significantly to the increased wall nucleation energy, whereas for higher thicknesses, the wall nucleation energy and nucleation field decrease, as the experimental results show and as was demonstrated, for example, for PLD Co thin films [35]. 

Consequently, to discuss our charged zigzag magnetic domain wall observations in these nanostructured Fe PLD films, all of these facts must be considered.

**Figure 9:**
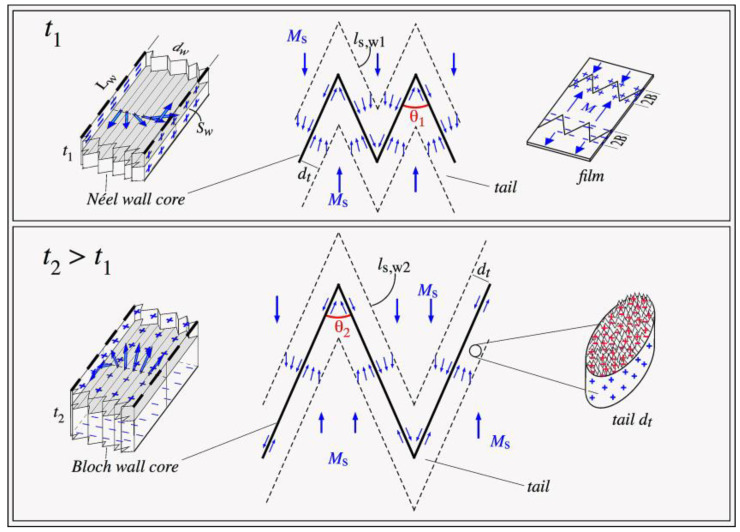
Schematic of two saw-tooth walls for Fe films with thicknesses *t*_1_ < *t*_2_. Walls are composed of a core and a tail. Wall angles were θ_1_ and θ_2_, respectively, with θ_1_ > θ_2_, as our experimental results revealed; segment lengths were *l*_s,w1_ and *l*_s,w2_, with *l*_s,w1_ < *l*_s,w2_, also according to the experimental results. At low thickness, the core had a Néel-type wall, with magnetization rotation within the wall in the film plane. At higher thickness, it had a Bloch-type wall, with magnetization out-of-plane rotation. Details of the two cores are shown (left side). Core wall width was *d*_w_ and tail width was *d*_t_, which were assumed to be thickness independent; see text for explanation. Magnetization rotation in the tail was also produced in the film plane. Representation of surface roughness with corresponding poles at the wall tail is shown on the right.

According to our experimental results (shown in Figure 5), we considered that the film with thickness *t*_1_, as demonstrated in Figure 9, had zigzag wall segments with length *l*_s,w1_ and the film with thickness *t*_2_ > *t*_1_ had length *l*_s,w2_, where *l*_s,w2_ > *l*_s,w1_. Similar to the spine model [28,29,30], there was a region with Néel-type tails *d*_t_ wide with an in-plane magnetic moment rotation; thus, there was a non-zero divergence of ***M***, ∇**M** ≠ 0, and positive and negative magnetic poles as in Néel magnetic walls [28,29,30]. The wall core was *d*_w_ wide, which was assumed to be equal for all of the films, independent of thickness *t*. This approximation is justified below.

Figure 9 shows a schematic of the effect of surface roughness on magnetization at the surface of the walls’ core and tail: at low thickness *t*_1_, the energy associated with roughness was dominant in the wall’s energetic balance because the magnetic moment carriers strongly interacted with the surface irregularities; at higher thickness *t*_2_, the effects of the surface morphology were smaller compared with the effect of the Néel tail’s volume charges.

The energies of the Néel (*E*_NW_) and Bloch (*E*_BW_) cores of the walls were calculated. For the calculations, the following data and parameters were considered:

(1) Experimental values of energy density corresponding to nucleation magnetic fields μ_o_*M*_s_*H*_N_.
(2) Experimental observation of crosstie walls in the 33 nm thick film.
(3) Observation of a cross-over from Néel walls (or crossties) to Bloch walls in the 55 nm thick film.
(4) Observation of Bloch lines and very weak crosstie walls in the 55 nm thick film.
(5) Observation of Bloch walls in the films thicker than 55 nm.
(6) Saturation magnetization μ_0_*M*_s_ = 2.0 T [13].
(7) Experimental θ values varying between ≈29° (for *t* = 11 nm) and 18° (for *t* = 82 nm).
(8) We also assumed an energy density term proportional to the roughness-to-thickness ratio *R*_q_/*t* that affected the whole wall, core, and tail. We considered this term according to our experimental *R*_q_/*t* values indicating that this ratio markedly decreased as the thickness increased and accounted for the additional energy contribution necessary for nucleation of the charged wall, which was Néel, crosstie, or Bloch type depending on *t* [28,30].

The total energy to produce nucleation of the magnetic walls and consequently the irreversible magnetization processes to saturate a film using the applied magnetic field (shown in Figure 3), expressed in J, is:

(1) $$\mu_{0}M_{s}H_{N}S_{T}t$$

where *M*_s_ is the film’s saturation magnetization, *H*_N_ is the nucleation applied magnetic field, *S*_T_ is the total film surface area, and *t* is the film thickness. This energy was used to nucleate the magnetic wall. This means the energy was

(2) $$\mu_{0}M_{s}H_{N}S_{T}t\;=\left[A\left(\frac{\pi^{2}}{d_{w}}\right)+\;\frac{1}{2}Kd_{w}+\;\frac{1}{4}\mu_{0}M_{s}^{2}\frac{t\;d_{w}}{d_{w}+t}\right]L_{w}t$$

for a Néel-type magnetic wall or

(3) $$\mu_{0}M_{s}H_{N}S_{T}t\;=\left[A\left(\frac{\pi^{2}}{d_{w}}\right)+\;\frac{1}{2}Kd_{w}+\;\frac{1}{4}\mu_{0}M_{s}^{2}\frac{d_{w}^{2}}{d_{w}+t}\right]L_{w}t$$

for a Bloch-type wall, where *A* is the exchange stiffness constant, *d*_w_ is the width of the magnetic wall, *K* is the crystalline energy density, and *L*_w_ is the total length of the nucleated magnetic wall. Using the experimental results shown in Figure 2 and Figure 3, we considered *K* ≈ 0.

Since we also considered a Néel-type wall tail and an energetic term whose origin was the interaction of the magnetization of the whole wall, core plus tail, with the roughness of the film surface, then [67]:

(4) $$\begin{array}{cc} \mu_{0}M_{s}H_{N}S_{T}t\; & =\{A\left[\left(\frac{\pi^{2}}{d_{w}}\right)+\;\left(\frac{{\left(\theta /2\right)}^{2}}{d_{t}}\right)\;\right]+\;\frac{1}{4}\mu_{0}M_{s}^{2}\left[\frac{t\;d_{w}}{d_{w}+t}+\;\left(\frac{2\left(1-\cos(\theta /2\right){)}^{2}\;t\;d_{t}}{t+d_{t}}\right)\right]\}L_{w}\\ & +\;\frac{1}{2}\mu_{0}M_{s}^{2}N_{d}L_{w}\left(d_{w}+\;2d_{t}\right)R_{q}\; \end{array}$$

and another similar equation for the Bloch core wall, where *A* is the exchange stiffness constant, *d*_w_ is the width of the magnetic wall, *L*_w_ is the total length of the nucleated magnetic wall, θ is the zigzag wall’s vertex angle, *d*_t_ is the width of the magnetic wall’s tail, and *N*_d_ is an effective demagnetizing factor that acts on the rough volume with surface area *L*_w_(*d*_w_ + 2 *d*_t_) and thickness *R*_q_, and *R*_q_ is the root mean square of the surface roughness, that is, the height of the roughness.

We used this equation to fit our experimental density energy values in J m^−3^ corresponding to the measured nucleation magnetic field *H*_N_ for different Fe films with magnetization μ_0_*M*_s_ and varying thickness *t*, as shown in Figure 4. This was:

(5) $$\begin{array}{cc} \mu_{0}M_{s}H_{N}= & \{A\left[\left(\frac{\pi^{2}}{d_{w}}\right)+\;\left(\frac{{\left(\theta /2\right)}^{2}}{d_{t}}\right)\;\right]+\;\frac{1}{4}\mu_{0}M_{s}^{2}\left[\frac{t\;d_{w}}{d_{w}+t}+\;\left(\frac{2\left(1-\cos(\theta /2\right){)}^{2}\;t\;d_{t}}{t+d_{t}}\right)\right]\}\frac{L_{W}}{S_{T}}\\ & +\;\frac{1}{2}\mu_{0}M_{s}^{2}N_{d}R_{q}\frac{L_{w}}{S_{T}}\left(\frac{d_{w}+2d_{t}}{t}\right) \end{array}$$

in the region with different *t* values corresponding to the Neel-type core wall and another similar equation for *t* values after the crossover, approximately *t_crossover_* = *d*_w_, from Neel to Bloch core magnetic walls.

For these reasons and from the films’ experimental behavior, we selected *d*_w_ = 55 nm as the most appropriate value. As demonstrated in Figure 5, we found an upper limit for the parameter *L*_w_/*S*_T_, that is, a factor in the expression (1/4)μ_0_*M*_s_^2^(L_w_/*S*_T_) must be considered; a factor of 4π and the best fitting data had a value (*L*_w_/*S*_T_) = 2500 m^−1^ that was higher than that obtained from the experimental images, ≈2000 m^−1^. For that, we used a factor of 16.3, which gave a value of *L*_w_/*S*_T_ = 2000 m^−1^. Thus, the total length of the nucleated zigzag magnetic wall per unit of surface area of the sample ≤ 2.0 mm/mm^2^ was deduced. Since the medium value of the surface roughness *R*_q_ was ≈2 nm, if a surface roughness demagnetizing factor of approximately *N*_d_ ≈ 0.2 was assumed, it was possible to establish the width of the tail of the zigzag nucleated magnetic wall, *d*_t_ ≈ 5500 nm = 100 *d*_w_. This tail width value was not so high compared with the length of the zigzag wall’s segments, which varied between *l*_s,w_ ≈ 300 μm and 2000 μm.

**Figure 10:**
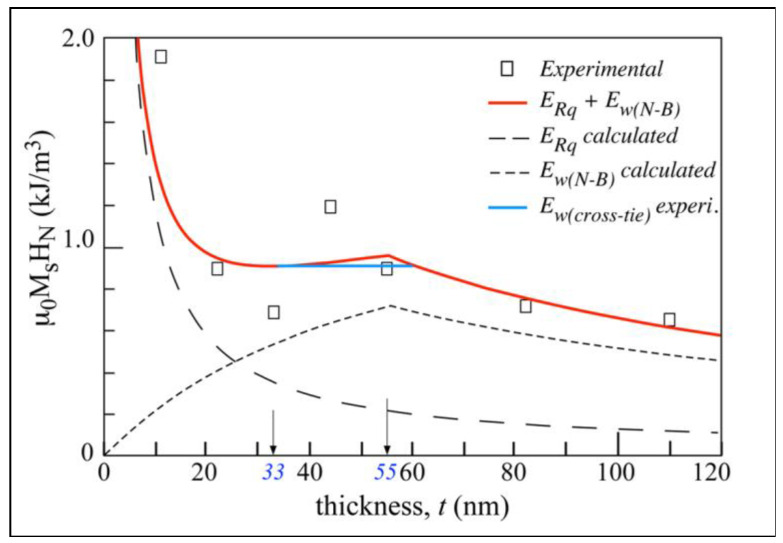
Fit (solid red line) of experimental values (square symbols) of applied magnetic field to nucleate magnetic domain walls using expression of Néel or Bloch wall energy, *E*_w(N-B),_ plus energy corresponding to magnetostatic interaction of core plus wall tail with surface roughness of sample *E*_Rq_. Lower crosstie wall energy *E*_w(crosstie)_ is illustrated for the interval of sample thickness between 33 nm and 55–60 nm. Parameter values used in the fit are: exchange stiffness constant *A* = 1.8 × 10^–11^ J m^–1^, magnetic anisotropy constant *K* ≈ 0, magnetization μ_0_*M*_s_ = 2 T, wall thickness *d*_w_ = 55 nm, ratio of total length of nucleated magnetic wall to total film surface area *L*_w_/*S*_T_ = 2.0 mm^−1^, surface roughness *R*_q_ = 2 nm, demagnetizing factor *N*_d_ = 0.2, and tail of charged wall *d*_t_ = 5.5 μm.

Figure 10 corresponds to the fit of our samples with the previously described criterion and the following parameters: exchange stiffness constant *A* = 1.8 × 10^−11^ J m^−1^, magnetic anisotropy constant *K* ≈ 0, magnetization μ_0_*M*_s_ = 2 T, wall thickness *d*_w_ = 55 nm, ratio of total length of nucleated magnetic wall to total film surface area *L*_w_/*S*_T_ = 2.0 mm^−1^, surface roughness *R*_q_ = 2 nm, demagnetizing factor *N*_d_ = 0.2, and tail of charged wall *d*_t_ = 5.5 μm. It also included an energy corresponding to the crosstie core walls at thicknesses between *t* ≈ 30 and 60 nm [68] and in this range of thicknesses with lower values than those associated with Néel or Bloch cores.

**Figure 11:**
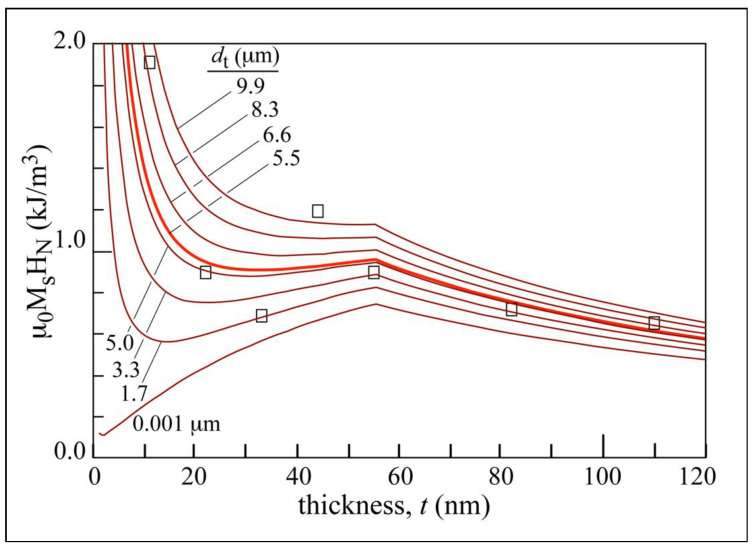
Set of calculated density energy curves (Equation (5)) for different values of tail wall width parameter *d*_t_. The case where the effect of surface roughness is almost null corresponds to the absence of this tail *d*_t_ = 1 nm. Experimental values of the applied magnetic field to nucleate walls (square symbols) are represented as well as its best fit (bold red line) for *d*_t_ = 5500 nm.

Figure 11 shows the effect of surface roughness on the wall’s magnetostatic energy, and more specifically on the wall’s tail. Figure 11 shows the calculated curves corresponding to the dependence on film thickness of the nucleation magnetic field energy using Equation (5) and the tail wall width parameter *d*_t_. The case where the effect of surface roughness is almost null corresponds to the absence of this tail *d*_t_ = 1 nm, which clearly did not fit our experimental data corresponding to the nucleation magnetic field. The influence of surface roughness on these charged walls is proven here.

It is seen that for these Fe films of low thickness, the energy of the wall mainly originated by the surface roughness interacting with its Néel-type core. This energy was always lower than the energy corresponding to the Bloch-type core. The energy due to roughness predominated in the behavior of *H*_N_, although the energy of the Néel wall was small. This was the reason why the zigzag walls were so sinuous for Fe films 11 nm thick, minimizing the effect of surface roughness.

Summarizing, we have experimentally shown the evolution with thickness of charged magnetic domain walls of Fe films, and specifically the first experimental evidence of the crossover between Néel-type and Bloch-type cores of these Fe-charged walls with the presence of crosstie walls. We studied the influence of surface roughness on these magnetic structures, and considering the different magnetic energies involved, an energetic fit to the nucleation magnetic field values was accomplished.

Previous studies showed the presence of charged crosstie walls in 50 nm thick single-crystal Ni films [69], 35 nm thick permalloy films [70], 42 nm thick Co films [28], and Fe films between 20 and 60 nm thick [71], but no evolution with thickness was demonstrated. All of these materials exhibited lower saturation magnetization values at room temperature than Fe films, so Fe films present an advantage for technological applications.

## 4. Conclusions

The magnetic domain wall structures of soft magnetic nanostructured Fe thin films were investigated and the charged zigzag magnetic domain walls were visualized. Changes in the core type of the saw-tooth walls were observed when the thickness of the films changed: at low thickness, up to ≈ 30 nm, the Néel core was visualized. In 33 nm thick samples, walls with crosstie cores were observed. For films with thickness greater than 55 nm, Bloch-type cores were present. This is the first time that this transition of the core of charged zigzag walls with the formation of crosstie walls has been shown in charged walls of amorphous or nanocrystalline Fe thin films.

These micromagnetic domain wall structures were linked to surface roughness. We associated the changes in nucleation magnetic field values corresponding to zigzag walls not only with the walls’ magnetostatic energy (corresponding to “flat” samples) but also with the decisive influence of the surface roughness energy, considering the extra magnetostatic energy originated by this roughness and the energies corresponding to Néel and Bloch walls.
